# Supplementary material for: Vascular smooth muscle cell loss, but not neuroinflammation, drives cerebrovascular reactivity impairment in Alzheimer’s disease
Source: bioRxiv. 2025 Dec 3:2025.12.01.691642. Preprint. [Version 1] doi: 10.64898/2025.12.01.691642 (PMC12822721; doi:10.64898/2025.12.01.691642)
Supplement: Supplementary file 1 [file media-1.pdf]

Vascular smooth muscle cell loss, but not neuroinflammation, drives cerebrovascular reactivity impairment in Alzheimer's disease

Xiuli Yang, Yuguo Li, Minmin Yao, Adnan Bibic, Wenzhen Duan, Hanzhang Lu, Zhiliang Wei

Supporting Information

1. Cerebral blood flow (CBF) and cerebrovascular reactivity (CVR) in 5xFAD mice

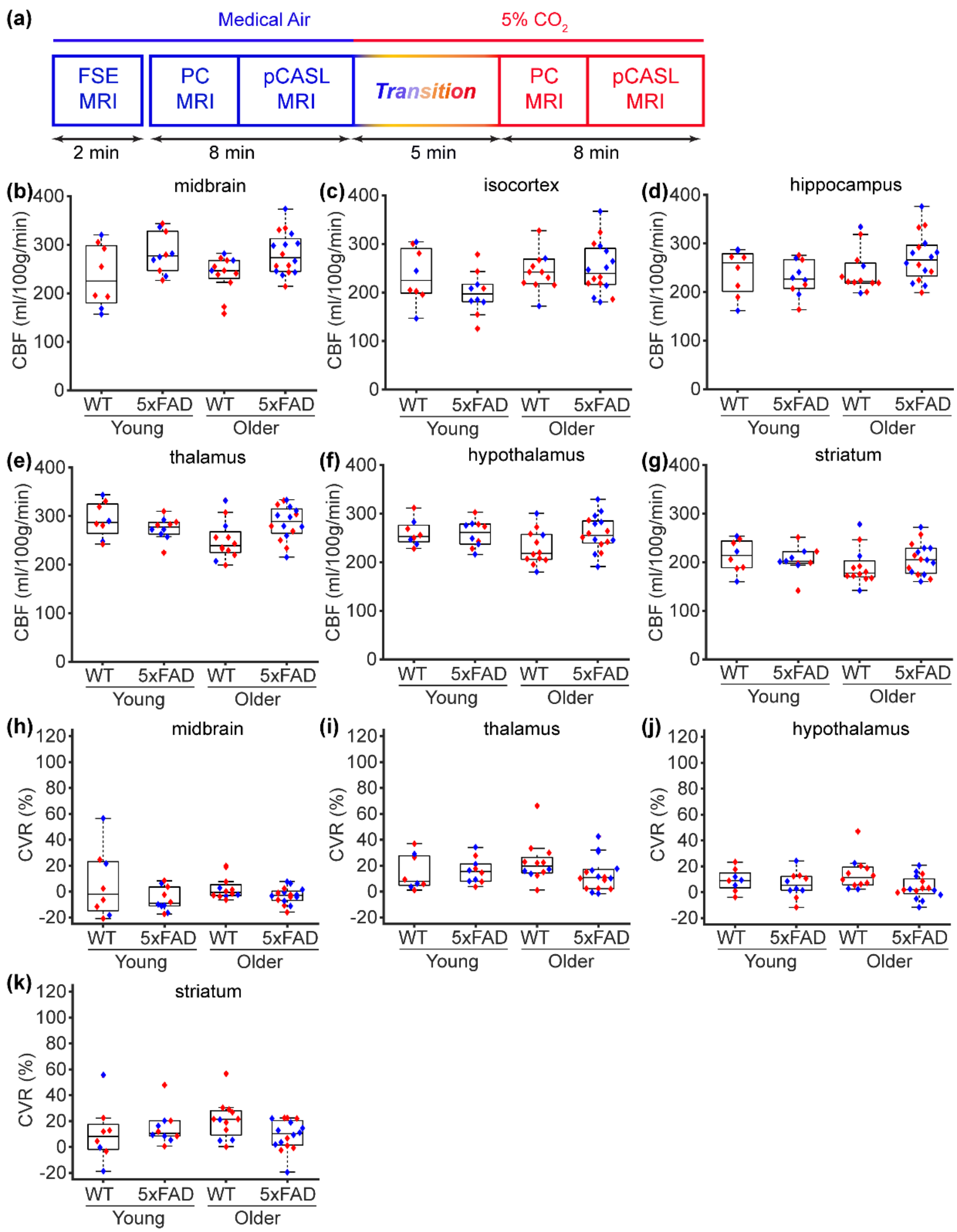

Figure S1 Regional cerebral blood flow (CBF) and cerebrovascular reactivity (CVR) values in young and older 5xFAD mice. (a) Schematic diagram of the experimental design. Under medical air, a fast spin-echo (FSE) MRI sequence was used to estimate brain volume, and both phase-contrast (PC) and pseudo-continuous arterial spin labeling (pCASL) MRI were performed to measure baseline CBF. After switching to 5% CO2 gas, a 5-min transition period was used, after which PC and pCASL scans were repeated to assess regional CVR. (b–g) Regional CBF values in the midbrain, isocortex, hippocampus, thalamus, hypothalamus, and striatum, respectively. (h–k) Regional CVR values in the midbrain, thalamus, hypothalamus, and striatum, respectively.

2. Immunofluorescent images of α-smooth muscle actin (α-SMA) and Collagen IV staining in 5xFAD mice

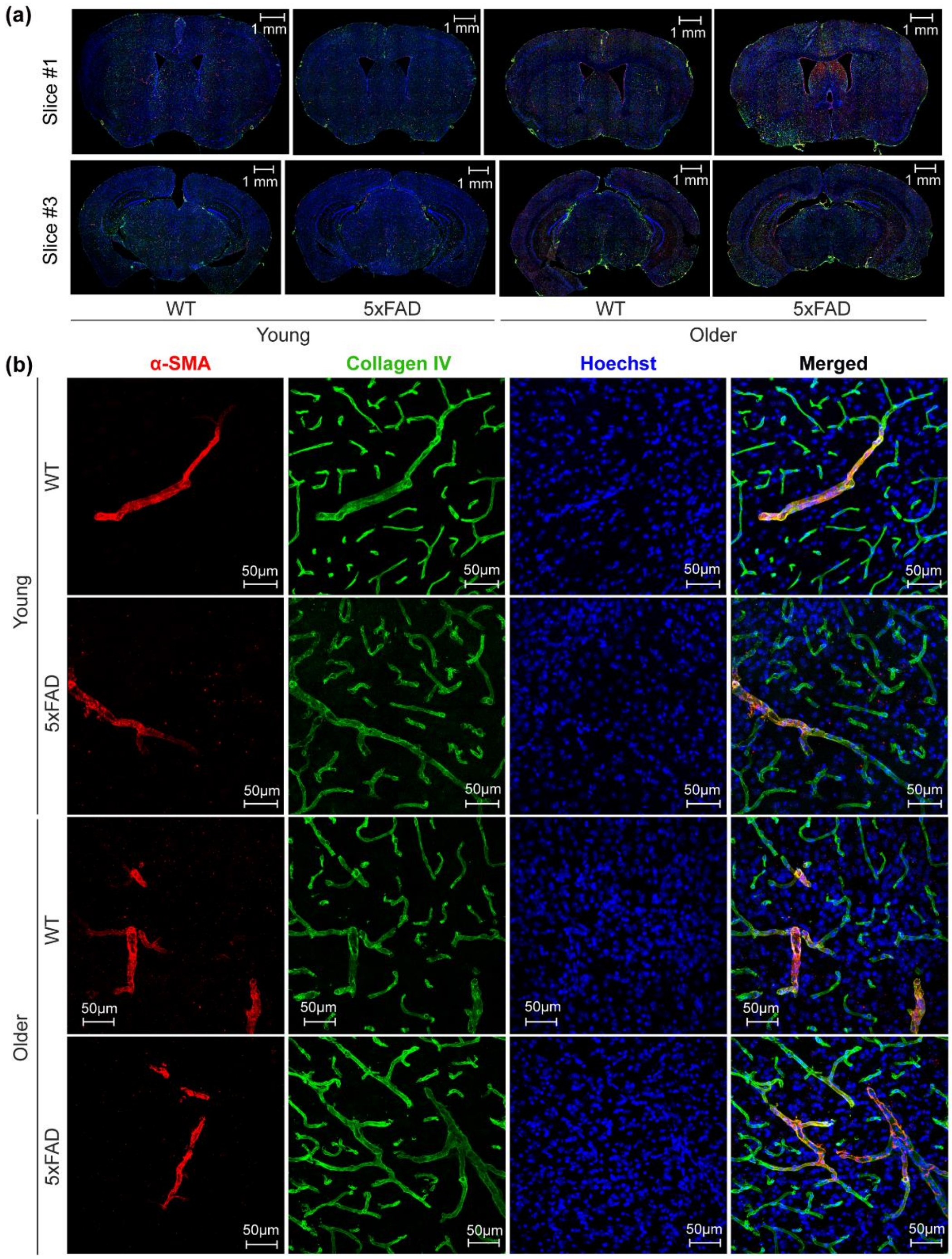

Figure S2 (a) Whole-slice and (b) isocortical immunofluorescent images showing α-SMA (red) and Collagen IV (green) staining. Hoechst staining (blue) was used to visualize nuclei as a reference. Slice #1 to #3 indicate the rostral-to-caudal direction. Scale bars: 1 mm (whole slice) and 50 µm (isocortex).

3. Regional CBF and CVR in cerebral autosomal dominant arteriopathy with subcortical infarcts and leukoencephalopathy (CADASIL) mice

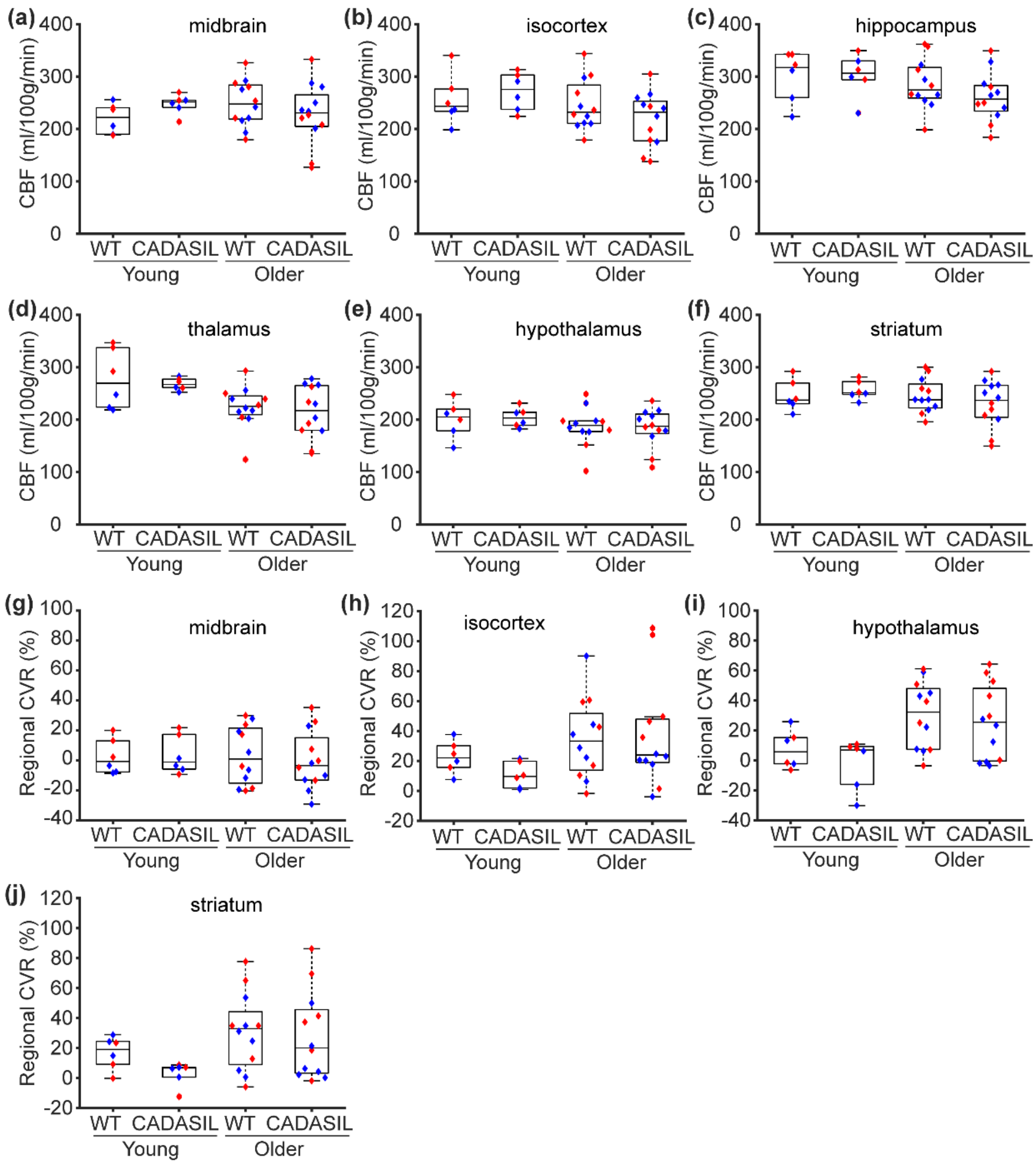

Figure S3 Regional cerebral blood flow (CBF) and cerebrovascular reactivity (CVR) values in young and older cerebral autosomal dominant arteriopathy with subcortical infarcts and leukoencephalopathy (CADASIL) mice. (a–f) Regional CBF values in the midbrain, isocortex, hippocampus, thalamus, hypothalamus, and striatum, respectively. (g–j) Regional CVR values in the midbrain, isocortex, hypothalamus, and striatum, respectively.

4. Immunofluorescent images of α-SMA and Collagen IV staining in the thalamus region of CADASIL mice

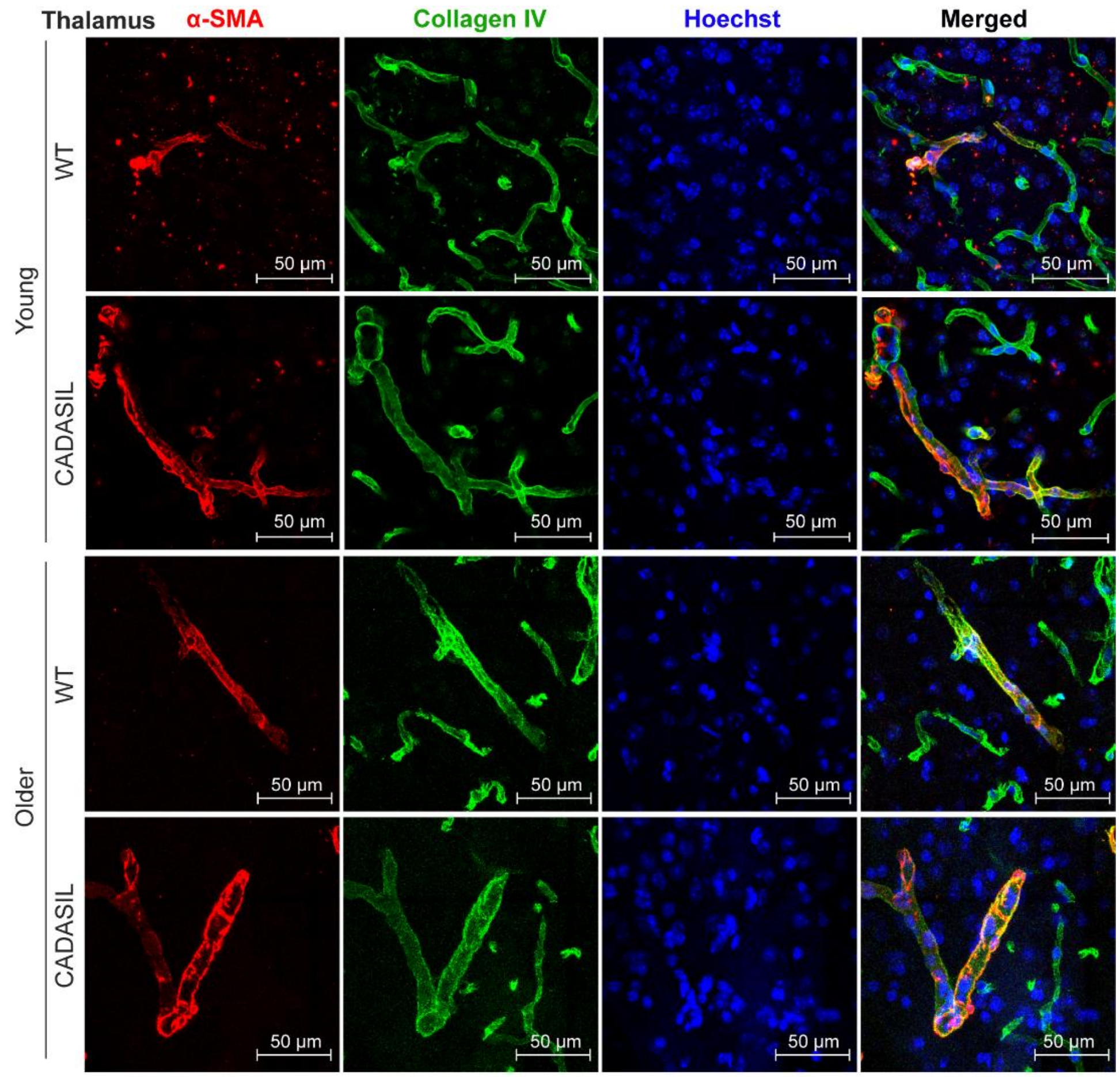

Figure S4 Immunofluorescent images of α-smooth muscle actin (α-SMA; red) and Collagen IV (green) staining in the thalamus of cerebral autosomal dominant arteriopathy with subcortical infarcts and leukoencephalopathy (CADASIL) mice. Hoechst staining (blue) was used to visualize nuclei. Scale bar: 50 μm.

5. Regional CBF and CVR in lipopolysaccharide (LPS)-induced neuroinflammation model

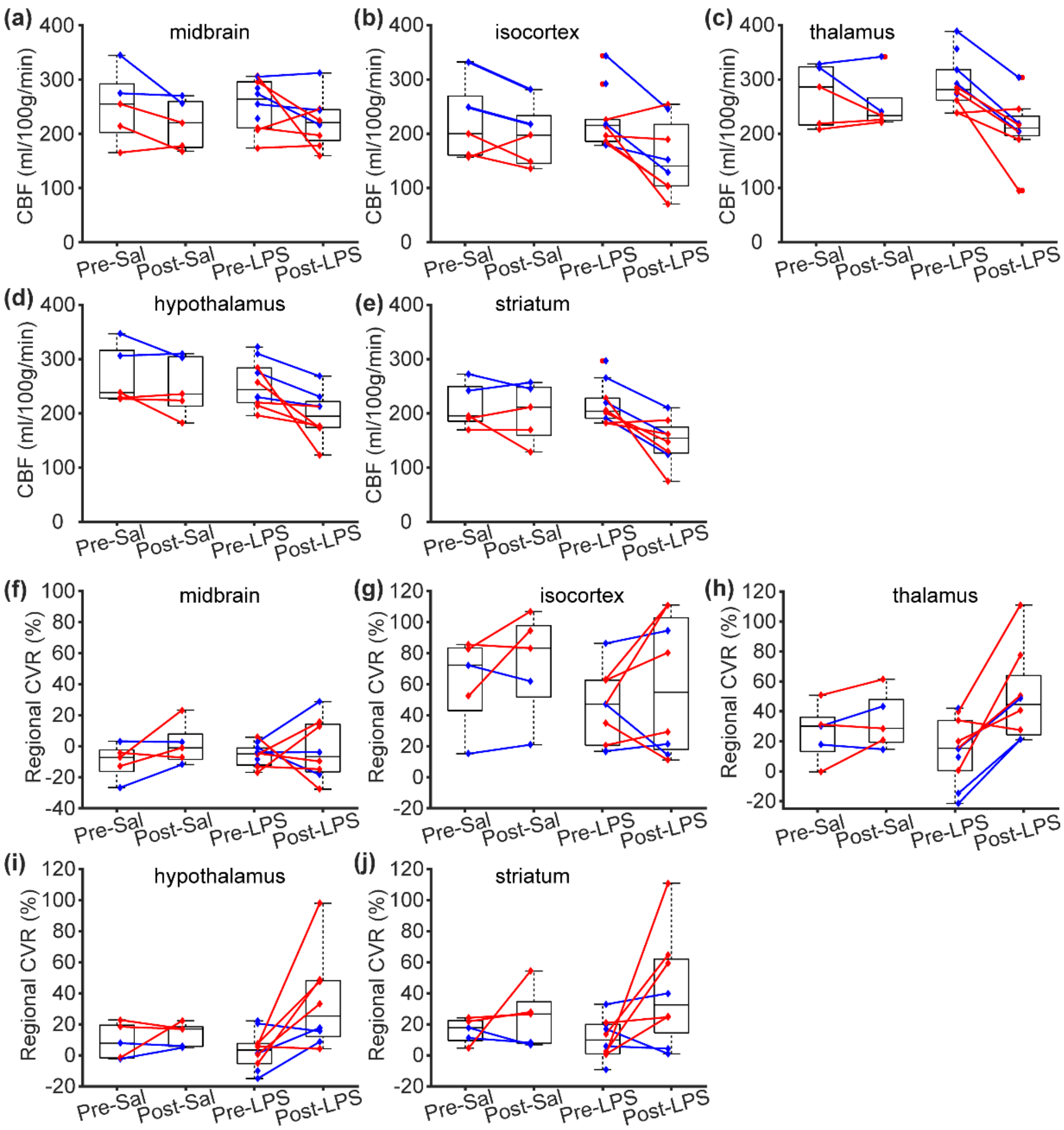

Figure S5 Regional cerebral blood flow (CBF) and cerebrovascular reactivity (CVR) values before and after saline or lipopolysaccharide (LPS) injection. (a–e) Regional CBF values in the midbrain, isocortex, thalamus, hypothalamus, and striatum at pre-saline (Pre-Sal), post-saline (Post-Sal), pre-LPS, and post-LPS conditions. (f–j) Corresponding regional CVR values in the midbrain, isocortex, thalamus, hypothalamus, and striatum.

6. Immunofluorescent images of ionized calcium-binding adaptor molecule 1 (Iba1) and CD68 staining in the neuroinflammation and 5xFAD models

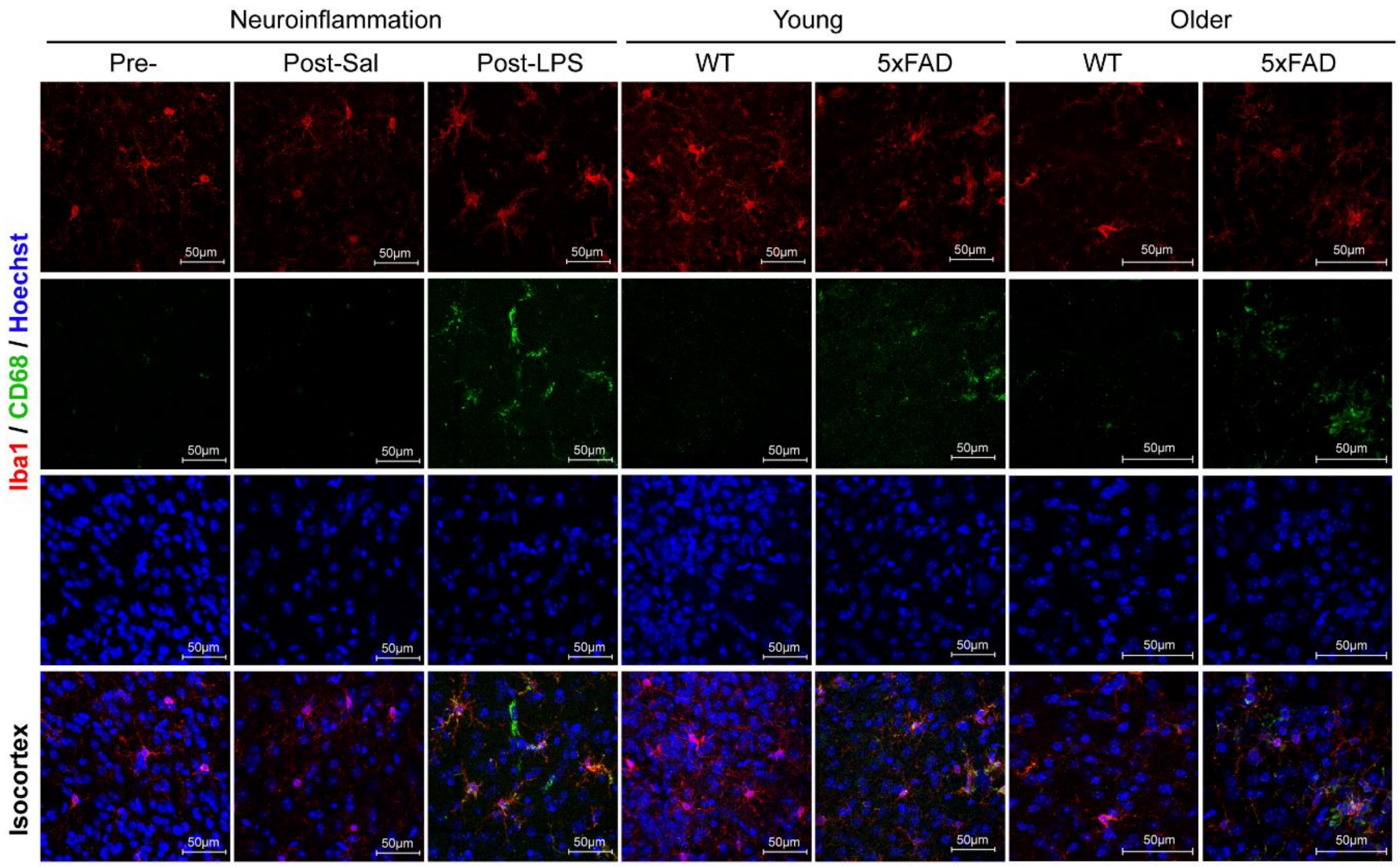

Figure S6 Representative immunofluorescent images showing ionized calcium-binding adaptor molecule 1 (Iba1; red) and CD68 (green) staining in the isocortex of lipopolysaccharide (LPS)-induced neuroinflammation and 5xFAD mice. Hoechst staining (blue) was used to visualize nuclei. Scale bar: 50 μm.

7. Immunofluorescent images of α-SMA and Collagen IV staining in the neuroinflammation model

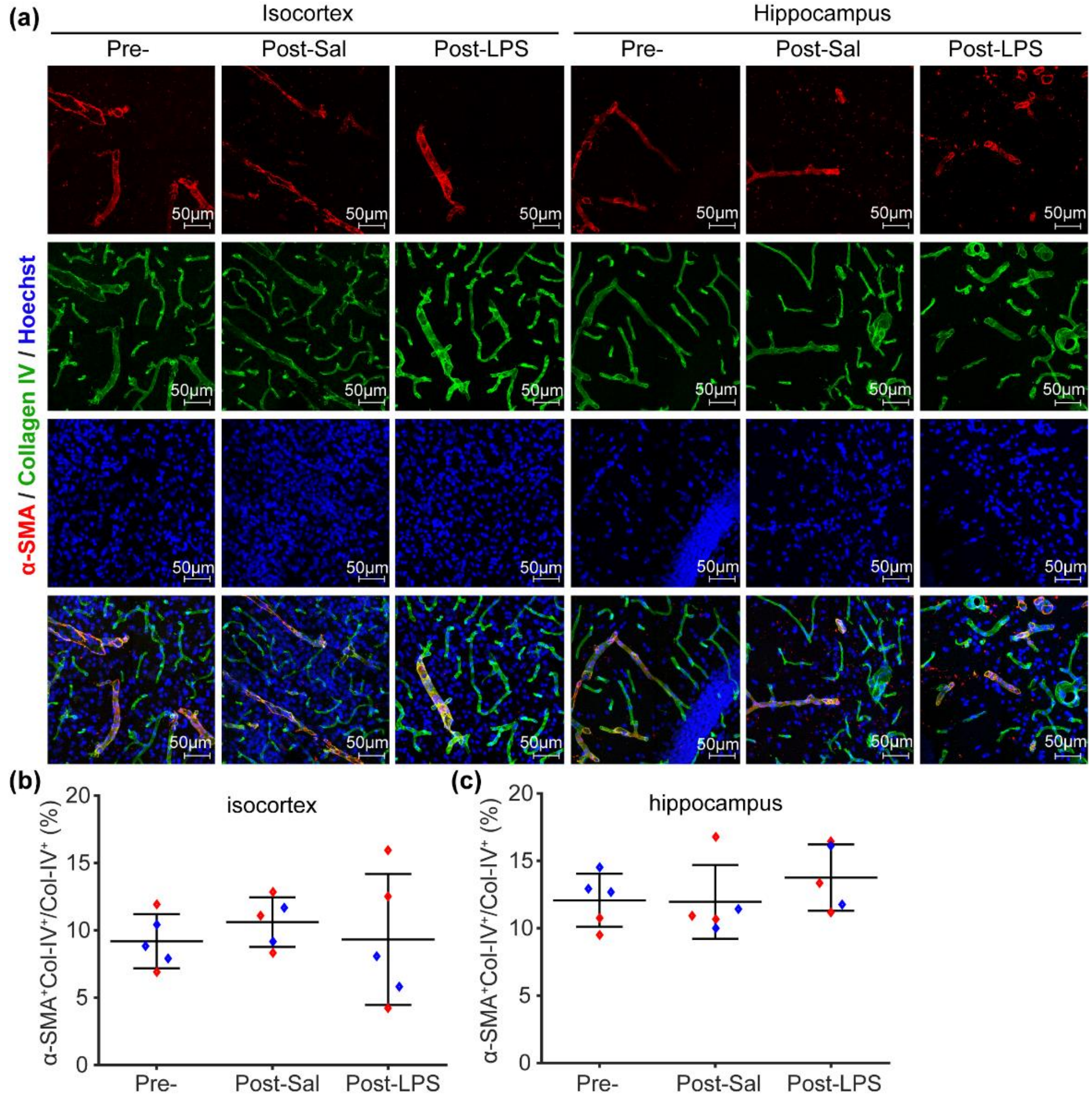

Figure S7 α-smooth muscle actin (α-SMA) coverage in the neuroinflammation model. (a) Representative immunofluorescent images showing α-smooth muscle actin (α-SMA; red) and Collagen IV (Col-IV; green) staining under pre-injection (Pre-), post-saline (Post-Sal), and post-lipopolysaccharide (Post-LPS) conditions in the isocortex and hippocampus. Hoechst staining (blue) was used to visualize nuclei. Scale bar: 50 µm. (b, c) α-SMA coverage index in the isocortex and hippocampus, respectively, across the examined conditions. The α-SMA coverage index is defined as the ratio of the co-localized α-SMA– and Collagen IV–positive area ($\alpha\text{-SMA}^{+}\text{Col-IV}^{+}$) to the total Collagen IV–positive area (Col-IV^{+}). Red and blue dots represent female and male mice, respectively. Error bars indicate standard deviation.

8. Detailed information of antibodies used in this study (Table S1)

#	Name	Vendor	Catalog #	Host	Feature	Conc. (μg/mL)	Pack volume(μL)	Optimal Dilution	Storage (˚C)
1	Collagen IV	Bio-Rad	2150-1470	Rabbit	Poly	-	100	1/500	-20
2	α-SMA	Sigma-Aldrich	C6198-.2ML	Mouse	Mono	-	200	1/250	4
3	Iba1	FujiFilm	019-19741	Rabbit	-	500-700	50	1/500	-20
4	CD68	Bio-Rad	MCA1957	Rat	Mono	1000	250	1/250	-20
5	6E10	Biolegend	803001	Mouse	Mono	1000	200	1/500	4

9. Details of statistical comparisons in this study (Table S2)

Comparison	Statistical information	*p*-value	Statistical information	*p*-value	Statistical information	*p*-value
Session 1. Vascular and metabolic responses to hypercapnia						
Student's *t*-test: CBF_{air} vs. CBF_{CO2}	$t[11] = -4.547$	8.346E-04				
t-test: OEF_{air} vs. OEF_{CO2}	$t[11] = 2.665$	0.022				
t-test: $CMRO2_{air}$ vs. $CMRO2_{CO2}$	$t[11] = -0.176$	0.864				
Pearson correlation: CBF vs. CVR	$R^2 = 0.740$	2.128E-17				
Session 2. Characterization of 5xFAD model[a]						
Brain volume ~ genotype + age + sex	$\beta_{genotype}$=-1.76E-03	0.699	β_{age}=5.65E-03	1.734E-08	β_{sex}=-4.82E-06	0.999
Global CBF ~ genotype + age + sex + rr	$\beta_{genotype}$=-3.43	0.745	β_{age}=-1.03	0.579	β_{sex}=-11.40	0.247
CVR ~ genotype * age + sex + CBF	$\beta_{genotype*age}$=-2.68	0.036	-	-	β_{sex}=0.67	0.841
CBF_{MB} ~ genotype + age + sex + rr	$\beta_{genotype}$=45.56	0.005	β_{age}=-0.02	0.994	β_{sex}=-4.50	0.756
CBF_{ISO} ~ genotype + age + sex + rr	$\beta_{genotype}$=-31.33	0.045	β_{age}=2.30	0.392	β_{sex}=-11.39	0.420
CBF_{HPC} ~ genotype + age + sex + rr	$\beta_{genotype}$=2.96	0.848	β_{age}=1.40	0.607	β_{sex}=-13.77	0.338
CBF_{TH} ~ genotype * age + sex + rr	$\beta_{genotype*age}$=8.83	0.022	-	-	β_{sex}=-9.01	0.375
CBF_{HY} ~ genotype + age + sex + rr	$\beta_{genotype}$=2.90	0.781	β_{age}=-4.51	0.018	β_{sex}=-7.77	0.423
CBF_{STR} ~ genotype + age + sex + rr	$\beta_{genotype}$=-7.18	0.500	β_{age}=-3.47	0.069	β_{sex}=-9.07	0.358
CVR_{MB} ~ genotype + age + sex + CBF_{MB}	$\beta_{genotype}$=-8.21	0.064	β_{age}=0.17	0.828	β_{sex}=-3.64	0.371
CVR_{ISO} ~ genotype * age + sex + CBF_{ISO}	$\beta_{genotype*age}$=-6.53	0.027	-	-	β_{sex}=-2.20	0.775
CVR_{HPC} ~ genotype * age + sex + CBF_{HPC}	$\beta_{genotype*age}$=-3.29	0.033	-	-	β_{sex}=-0.48	0.905
CVR_{TH} ~ genotype + age + sex + CBF_{TH}	$\beta_{genotype}$=-6.80	0.134	β_{age}=0.23	0.771	β_{sex}=-1.45	0.729
CVR_{HY} ~ genotype + age + sex + CBF_{HY}	$\beta_{genotype}$=-6.70	0.054	β_{age}=0.45	0.460	β_{sex}=1.27	0.691
CVR_{STR} ~ genotype + age + sex + CBF_{STR}	$\beta_{genotype}$=-4.07	0.430	β_{age}=0.36	0.697	β_{sex}=5.20	0.282
Young CVR_{HPC} ~ genotype + sex + CBF_{HPC}	$\beta_{genotype}$=4.64	0.598	-	-	β_{sex}=-2.68	0.740
Older CVR_{HPC} ~ genotype + sex + CBF_{HPC}	$\beta_{genotype}$=-17.05	3.064E-04	-	-	β_{sex}= 2.04	0.607
$CI(\alpha\text{-SMA/Col-IV})_{ISO}$ ~ genotype * age + sex	$\beta_{genotype*age}$=-0.20	0.023	-	-	β_{sex}=0.20	0.843
$CI(\alpha\text{-SMA/Col-IV})_{HPC}$ ~ genotype * age + sex	$\beta_{genotype*age}$=-0.23	0.024	-	-	β_{sex}=-0.88	0.440
t-test: Young WT vs. 5xFAD CI(α-SMA/Col-	$t[8] = 1.833$	0.104				

IV)$_{ISO}$						
t-test: Older WT vs. 5xFAD CI(α-SMA/Col-IV)$_{ISO}$	*t*[8] = 4.263	0.003				
t-test: Young WT vs. 5xFAD CI(α-SMA/Col-IV)$_{HPC}$	*t*[8] = 0.731	0.485				
t-test: Older WT vs. 5xFAD CI(α-SMA/Col-IV)$_{HPC}$	*t*[8] = 3.213	0.012				
CI(6E10/Region)$_{ISO}$ ~ genotype * age + sex	$\beta_{genotype*age}$=0.07	0.001	-	-	β_{sex}=-0.19	0.415
CI(6E10/Region)$_{HPC}$ ~ genotype * age + sex	$\beta_{genotype*age}$=0.10	6.710E-04	-	-	β_{sex}=-0.36	0.234
t-test: Young WT vs. 5xFAD CI(6E10/Region)$_{ISO}$	*t*[8] = -9.16	1.625E-05				
t-test: Older WT vs. 5xFAD CI(6E10/Region)$_{ISO}$	*t*[8] = -5.75	4.274E-04				
t-test: Young WT vs. 5xFAD CI(6E10/Region)$_{HPC}$	*t*[8] = -7.41	7.516E-05				
t-test: Older WT vs. 5xFAD CI(6E10/Region)$_{HPC}$	*t*[8] = -5.99	3.281E-04				
t-test: young vs. older CI(Col-IV/6E10)$_{ISO}$	*t*[8] = -2.677	0.028				
t-test: young vs. older CI(Col-IV/6E10)$_{HPC}$	*t*[8] = -5.179	8.435E-04				
CI(CD68/Iba1)$_{HPC}$ ~ genotype * age + sex	$\beta_{genotype*age}$= 0.86	0.004	-	-	β_{sex}=-2.92	0.351
CI(CD68/Iba1)$_{ISO}$ ~ genotype * age + sex	$\beta_{genotype*age}$= 0.90	0.002	-	-	β_{sex}=-0.74	0.794

Session 3. Characterization of CADASIL model

Brain volume ~ genotype + age + sex	$\beta_{genotype}$=-3.82E-03	0.498	β_{age}=-1.40E-03	0.255	β_{sex}=0.02	0.010
Global CBF ~ genotype + age + sex + rr	$\beta_{genotype}$=-4.03	0.744	β_{age}=-5.94	0.169	β_{sex}=0.23	0.985
CVR ~ genotype * age + sex + CBF	$\beta_{genotype}$=-6.49	0.024	β_{age}=1.88	0.004	β_{sex}=-7.87	0.007
CBF$_{MB}$ ~ genotype + age + sex + rr	$\beta_{genotype}$=-4.43	0.787	β_{age}=0.73	0.897	β_{sex}=6.94	0.668
CBF$_{ISO}$ ~ genotype + age + sex + rr	$\beta_{genotype}$=-10.18	0.553	β_{age}=-8.89	0.139	β_{sex}=-8.61	0.610
CBF$_{HPC}$ ~ genotype + age + sex + rr	$\beta_{genotype}$=-11.41	0.476	β_{age}=-9.36	0.096	β_{sex}=-15.62	0.324
CBF$_{TH}$ ~ genotype * age + sex + rr	$\beta_{genotype}$=-5.08	0.741	β_{age}=-15.22	0.007	β_{sex}=4.60	0.761
CBF$_{HY}$ ~ genotype + age + sex + rr	$\beta_{genotype}$=1.96	0.870	β_{age}=-5.72	0.174	β_{sex}=5.97	0.615
CBF$_{STR}$ ~ genotype + age + sex + rr	$\beta_{genotype}$=-4.03	0.744	β_{age}=-5.94	0.169	β_{sex}=0.23	0.985
CVR$_{MB}$ ~ genotype + age + sex + CBF$_{MB}$	$\beta_{genotype}$=-3.19	0.552	β_{age}=-0.47	0.686	β_{sex}=-11.77	0.033

CVR_{ISO} ~ genotype + age + sex + CBF_{ISO}	$\beta_{genotype}$=-6.85	0.191	β_{age}=2.58	0.028	β_{sex}=-14.30	0.009
CVR_{HPC} ~ genotype + age + sex + CBF_{HPC}	$\beta_{genotype}$=-4.32	0.226	β_{age}=1.85	0.022	β_{sex}=-7.40	0.042
CVR_{TH} ~ genotype + age + sex + CBF_{TH}	$\beta_{genotype}$=-9.75	0.026	β_{age}=3.01	0.002	β_{sex}=0.37	0.929
CVR_{HY} ~ genotype + age + sex + CBF_{HY}	$\beta_{genotype}$=-9.93	0.028	β_{age}=3.94	2.245E-04	β_{sex}=-14.51	0.002
CVR_{STR} ~ genotype + age + sex + CBF_{STR}	$\beta_{genotype}$=-10.49	0.030	β_{age}=2.59	0.016	β_{sex}=-12.29	0.012
$CI(\alpha\text{-SMA/Col-IV})_{HPC}$ ~ genotype + age + sex	$\beta_{genotype}$=-1.71	0.113	β_{age}=0.14	0.492	β_{sex}=-1.66	0.124
$CI(\alpha\text{-SMA/Col-IV})_{TH}$ ~ genotype + age + sex	$\beta_{genotype}$=-2.01	0.008	β_{age}=-0.17	0.229	β_{sex}=0.14	0.833

Session 4. Characterization of neuroinflammation model[b]

Brain volume ~ injection + sex + ε	β_{saline}=2.13E-03	0.775	β_{LPS}=-2.47E-03	0.696	β_{sex}=0.02	4.669E-04
Global CBF ~injection + sex + rr + ε	β_{saline}=-13.53	0.439	β_{LPS}=-39.46	0.039	β_{sex}=-62.75	5.464E-05
Global CVR ~ injection + sex + CBF + ε	β_{saline}=7.68	0.381	β_{LPS}=6.88	0.459	β_{sex}=1.73	0.848
CBF_{MB} ~ injection + sex + rr + ε	β_{saline}=-31.29	0.156	β_{LPS}=-21.05	0.357	β_{sex}=-56.84	0.001
CBF_{ISO} ~ injection + sex + rr + ε	β_{saline}=-25.79	0.350	β_{LPS}=-45.16	0.126	β_{sex}=-61.81	0.005
CBF_{HPC} ~ injection + sex + rr + ε	β_{saline}=-13.53	0.536	β_{LPS}=-20.90	0.365	β_{sex}=-62.34	6.895E-04
CBF_{TH} ~ injection + sex + rr + ε	β_{saline}=-31.24	0.152	β_{LPS}=-56.66	0.018	β_{sex}=-65.04	3.662E-04
CBF_{HY} ~ injection + sex + rr + ε	β_{saline}=-6.49	0.698	β_{LPS}=-38.91	0.035	β_{sex}=-63.12	3.024E-05
CBF_{STR} ~ injection + sex + rr + ε	β_{saline}=-13.15	0.457	β_{LPS}=-46.79	0.017	β_{sex}=-45.71	0.002
CVR_{MB} ~ injection + sex + CBF_{MB} + ε	β_{saline}=8.34	0.257	β_{LPS}=5.79	0.455	β_{sex}=0.42	0.956
CVR_{ISO} ~ injection + sex + CBF_{ISO} + ε	β_{saline}=17.09	0.252	β_{LPS}=-18.30	0.250	β_{sex}=-4.99	0.744
CVR_{HPC} ~ injection + sex + CBF_{HPC} + ε	β_{saline}=5.09	0.637	β_{LPS}=1.14	0.920	β_{sex}=-3.47	0.757
CVR_{TH} ~ injection + sex + CBF_{TH} + ε	β_{saline}=13.85	0.258	β_{LPS}=25.97	0.053	β_{sex}=12.17	0.337
CVR_{HY} ~ injection + sex + CBF_{HY} + ε	β_{saline}=5.38	0.552	β_{LPS}=17.81	0.074	β_{sex}=1.97	0.833
CVR_{STR} ~ injection + sex + CBF_{STR} + ε	β_{saline}=9.18	0.401	β_{LPS}=15.97	0.176	β_{sex}=2.26	0.841
$CI(CD68/Iba1)_{HPC}$ ~ injection + sex	β_{saline}=-0.91	0.511	β_{LPS}=6.67	4.437E-04	β_{sex}=0.67	0.559
$CI(CD68/Iba1)_{ISO}$ ~ injection + sex	β_{saline}=-0.19	0.794	β_{LPS}=5.91	4.224E-06	β_{sex}=0.76	0.215
$CI(\alpha\text{-SMA/Col-IV})_{HPC}$ ~ injection + sex	β_{saline}=-0.02	0.992	β_{LPS}=1.78	0.290	β_{sex}=-0.47	0.726
$CI(\alpha\text{-SMA/Col-IV})_{ISO}$ ~ injection + sex	β_{saline}=1.11	0.603	β_{LPS}=-0.18	0.932	β_{sex}=1.54	0.385

[a] rr: respiration rate; MB: midbrain; ISO: isocortex; HPC: hippocampus; TH: thalamus; HY: Hypothalamus; STR: striatum. $CI(\alpha\text{-SMA/Col-IV})_{ISO}$: Coverage index (CI) defined by the ratio between α-SMA^{+}Col-IV^{+} co-localization and Col-IV^{+} for the ISO region. Similar definitions followed the identical principle.

[b] Linear mixed-effects (LME) models were used for this part when relevant because repeated measurements in same mice were involved. A random term (ε) was included for each mouse in statistical analyses.
